# Supplementary material for: Immune-Deficient Pfp/Rag2−/− Mice Featured Higher Adipose Tissue Mass and Liver Lipid Accumulation with Growing Age than Wildtype C57BL/6N Mice
Source: Cells. 2019 Jul 25;8(8):775. doi: 10.3390/cells8080775 (PMC6721582; doi:10.3390/cells8080775)

Supplementary Materials

Table S1. List of proteins detected by the Proteome Profiler™ Mouse Adipokine Array Kit.

Adipokine	Alternative Name	Entrez Gene ID
Adiponectin	Acrp30/AidipoQ	11450
AgRP	ART	11604
ANGPT-L3	---	30924
C-reactive Protein	CRP	12944
DPPIV	CD26/DPP4	13482
Endocan	ESM-1	71690
Fetuin A	AHSG	11625
FGF acidic	FGF-1	14164
FGF-21	---	56636
HGF	---	15234
ICAM-1	CD54	15894
IGF-I	Somatomedin C	16000
IGF-II	Somatomedin A	16002
IGFBP-1	---	16006
IGFBP-2	---	16008
IGFBP-3	---	16009
IGFBP-5	---	16011
IGFBP-6	---	16012
IL-6	---	16193
IL-10	---	16153
IL-11	---	16156
Leptin	OB	16846
LIF	---	16878
Lipocalin-2	NGAL	16819
MCP-1	CCL2/JE	20296
M-CSF	CSF-1	12977
Oncostatin M	OSM	18413
Pentraxin 2	PTX2/SAP	20219
Pentraxin 3	PTX3/TSG-14	19288
Pref-1	DLK-1/FA1	13386
RAGE	---	11596
RANTES	CCL5	20304
RBP4	---	19662
Resistin	ADSF/FIZZ3	57264
Serpin E1	PAI-1	18787
TIMP-1	---	21857
TNF-α	TNFSF1A	21926
VEGF	VEGF-A	22339

Figure S1.

Graphical summary of serum parameters. Symbols over columns indicate statistical differences between groups at the p<0.05 level.

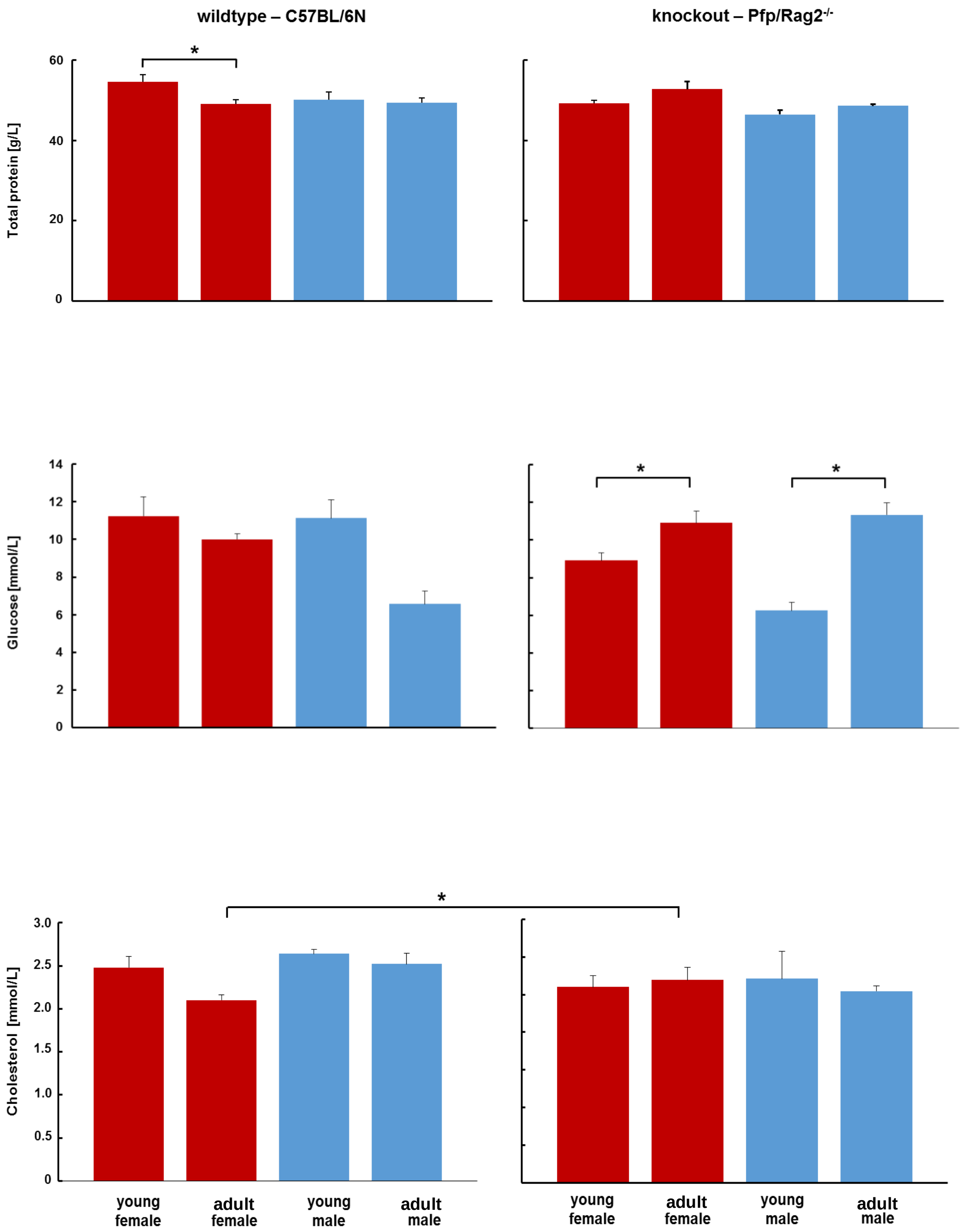

Figure S1, cont.

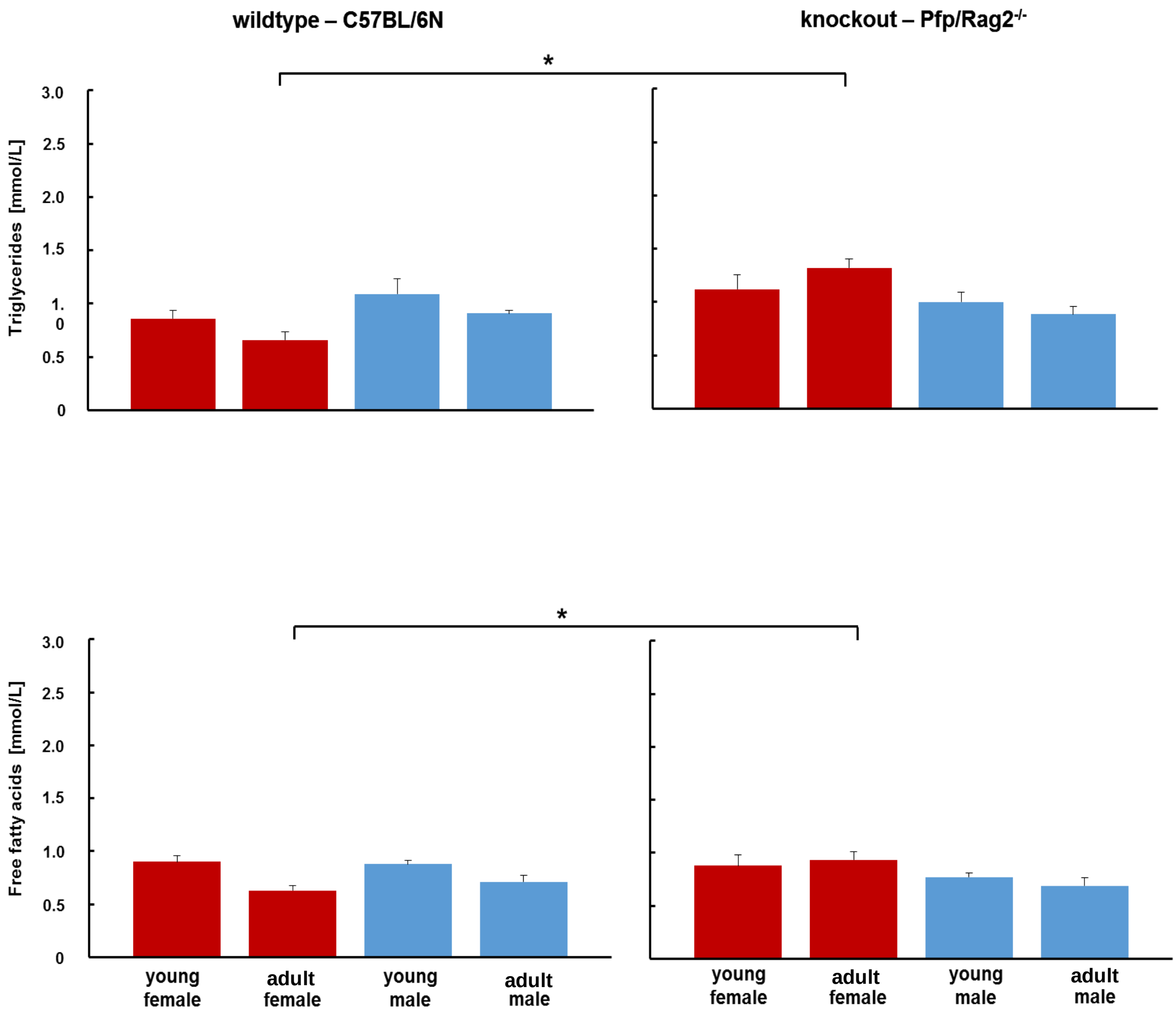

Figure S2. Zonal expression of periportal E-cadherin (E-cad) and of perivenous glutamine synthase (GS) and Cytochrome P450 2E1 (CYP2E1) in livers from wildtype and knockout female and male mice. In (a), a comprehensive scheme for the expression of the markers is given showing periportal E-cadherin and perivenous CYP2E1 as well as GS. (b) Immunofluorescent co-staining of E-cad (red) and GS (green) in the upper panels, and of E-cad (red) and CYP2E1 (green) in the lower panels of wildtype (left) and of knockout (right) female and male mice. Pictures are representative out of 3 different animals in each group. PV – portal vein; CV – central vein.

(a)

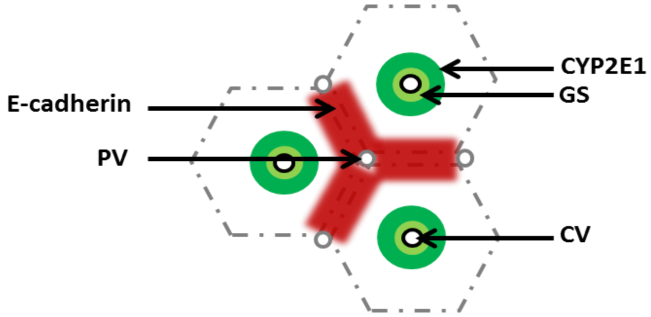

(b)

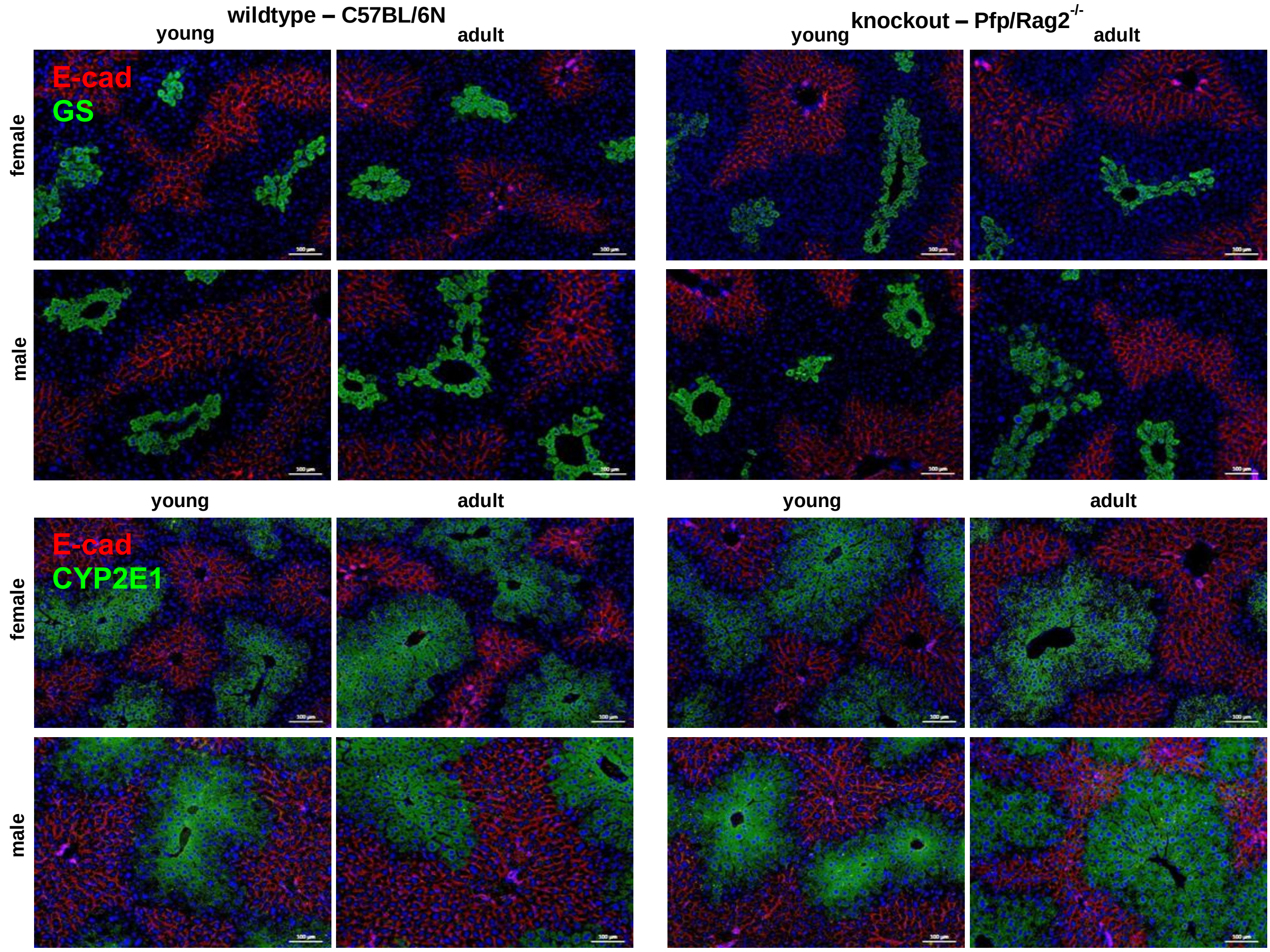

Figure S3. Expression levels of mRNA coding for proteins involved in lipid metabolism and function in isolated hepatocytes of wildtype and knockout female and male mice after 3 and 5 days of culture. Expression was quantified by semi-quantitative analysis of PCR products and the signals were normalized to the reference genes. Differences between groups were considered significant, if the p-value was ≤0.05 (denoted '*'; whereas p≤0.01, 0.001 and 0.0001 are denoted as '**', '***', and '****', respectively).

3 days of culture

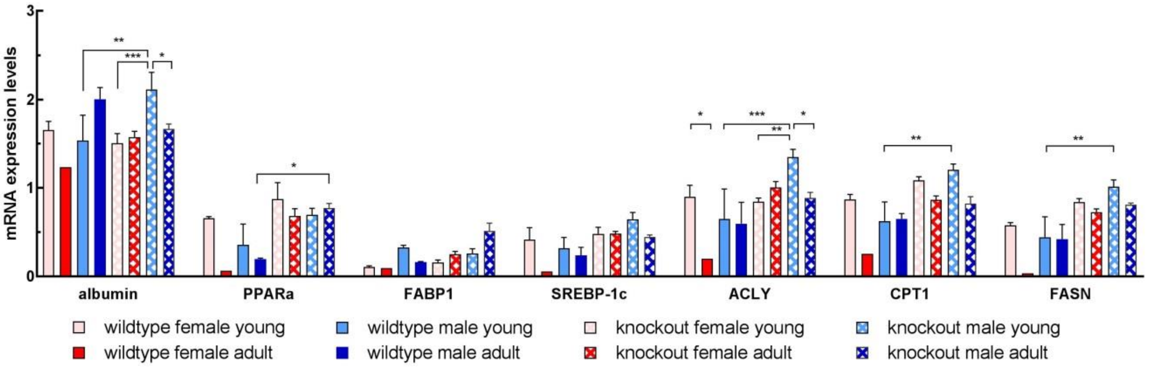

5 days of culture

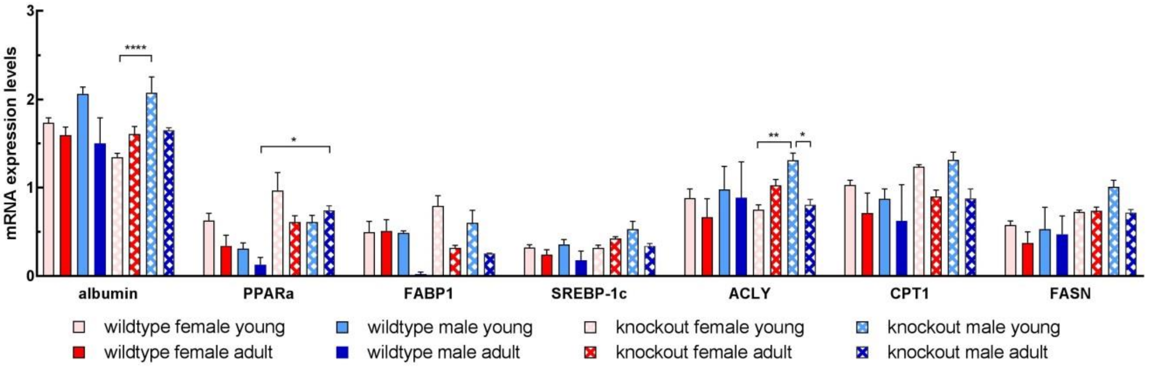

Supplement: Supplementary file 1 [file cells-08-00775-s001.pdf]
